# Treatment needs and skill mix workforce requirements for prosthodontic care: a comparison of estimates using normative and sociodental approaches

**DOI:** 10.1186/s12903-015-0015-9

**Published:** 2015-03-13

**Authors:** Norintan Ab-Murat, Aubrey Sheiham, Richard Watt, Georgios Tsakos

**Affiliations:** Department of Community Oral Health and Clinical Prevention, Faculty of Dentistry, University of Malaya, Kuala Lumpur, Malaysia; Department of Epidemiology and Public Health, University College London, London, United Kingdom

**Keywords:** Needs assessment, Prosthodontic treatment, Quality of life, Health workforce, Dental auxiliaries, Professional delegation

## Abstract

**Background:**

The traditional measure for assessing dental treatment needs and workforce requirements based solely on normative need (NN) has major shortcomings. The sociodental approach (SDA) to assess needs overcomes some of the shortcomings as it combines normative and subjective needs assessments and also incorporates behavioural propensity (Sheiham and Tsakos 2007).

The objective of this study was to estimate and compare prosthodontic treatment needs and workforce requirements, using the normative and the sociodental approaches for different skill mix models.

**Methods:**

A cross-sectional study was conducted on 732 university employees aged 30–54 years. Normative prosthodontic need was assessed using the WHO (1997) method. The SDA includes NN and also considers oral impacts, measured through the OIDP index, and behavioural propensity. Estimates of prosthodontic need and dental workforce requirements using the two methods were compared using McNemar and Wilcoxon Signed Rank test respectively. The dental workforce required for prosthodontic treatment based on NN and SDA approaches were then compared using different workforce skill mix models.

**Results:**

The proportion of subjects needing prosthodontic treatment was lower by more than 90% when the SDA was used compared to NN. The number of dentists required for prosthodontic treatment per 100,000 people were 98.8 using NN compared to 2.49 using SDA. Using a skill mix approach, the requirements for dentists per 100,000 people decreased slightly when more denture procedures were delegated to dental therapists.

**Conclusion:**

There were very much lower levels of prosthodontic treatment needs and workforce requirements when using the sociodental approach compared to normative methods.

## Background

The most common method of assessing dental treatment needs is the normative approach based on professional judgements. Despite its usefulness, the sole use of this approach has numerous shortcomings [1]. Using normative need alone when making clinical decisions may lead to overtreatment [2] and also give unrealistic estimates of workforce needs [3]. Good practice involves taking into account the perceived needs of patients as suggested in the sociodental approach [1].

The sociodental approach (SDA) is a comprehensive needs assessment model that integrates both normative and subjective measurements in assessing needs for dental care [1]. In the sociodental approach (SDA) model, subjective perceptions of need are measured using an oral health related quality of life (OHRQoL) indicator while normative assessments are obtained through clinical oral examination. In addition to normative and subjective measurements, the SDA also incorporates the assessment of people’s attitude and behaviour towards dental treatment and uses evidence-based dentistry to recommend effective treatments. There are two different models of the SDA. One is for people with dental diseases that progress chronically or threaten life, such as dental caries and oral cancer. Another is for people with non-life threatening and non-progressive dental conditions, such as periodontal disease and missing teeth [1]. For the former model, normative assessment takes precedence over subjective perceptions and appropriate dental treatments are provided based on the propensity to benefit. For the second model, all three main components of SDA, namely the normative assessment, subjective perceptions and behavioural propensity are assessed.

Studies comparing oral health needs using the normative (NN) and the SDA reported large differences in needs ranging from 40% to 90% [4-7]. However, only one study has so far converted those differences into dental workforce needs [6]. That study found a 78% lower need for workforce to treat prosthodontic needs using the SDA compared to the NN.

Oral health care delivery systems can only be cost-effective when provision of care is provided by those with most appropriate qualifications and skills [8]. Using a skill mix approach by utilising dentists and professionals complementary to dentistry (PCDs) to carry out dental tasks that correspond to their skill level increases accessibility, affordability and improve availability of services [9]. In some countries, there are laws permitting PCDs to provide dentures directly to patients. Such PCDs are called denturists in Canada, Finland and Denmark, clinical dental technicians in the UK and New Zealand and dental prosthetists in Australia [10]. Expanded-duty PCDs have been introduced in Malaysia, but only in the field of periodontology, orthodontics and paediatrics. These expanded-duty PCDs received further training in the respective clinical areas and are allowed to treat adults under indirect supervision of dentists at dental specialist clinics [11]. At present, expanded-duty PCDs have not yet been introduced in prosthodontics. The introduction of PCDs who are able to provide complete and partial dentures direct to the populations will provide a significant positive impact on the overall provision of oral health [12].

Throughout this paper, the term prosthodontic PCDs will be used to define dental auxiliaries legally allowed to provide and repair dentures directly to patients. Despite evidence showing that their technical ability is good [13] and they are highly accepted by patients [13,14], some dental organizations vigorously oppose using prosthodontic PCDs [15].

Only one study has compared dental needs and workforce estimates for prosthodontic treatment comparing NN and SDA among adults [6]. However, the study by Ryu et al. [6] which estimated prosthodontic workforce need using the SDA method, did not use a skill mix approach. The objective of this study was to compare prosthodontic treatment needs of a sample of Malaysian adults between the NN and SDA. We will also look at different skill mix scenarios that include the introduction of prosthodontic PCDs and compare the differences in the numbers of dentists and prosthodontic PCDs required.

## Methods

A cross-sectional study was carried out on a Malaysian adult sample of 30–54 year-old. The age range was chosen because adults aged 30–54 years have a fair number of missing teeth that may need replacement. The calculated sample size was 723, based on a predicted 40% difference in the prevalence of prosthodontic needs between normative and sociodental approach and a non-response of 10%. The sampling frame was university employees of a public university in Kuala Lumpur. All Malaysian employees in the selected university aged 30–54 years, and present in their offices during the survey, were invited to participate in this study. They were given an Information Sheet about the purpose and conduct of the survey and a Consent Form. Those who agreed to participate returned the Consent Form signed and dated, and it was also signed and dated by a witness to confirm that informed consent was provided. The survey contained a face-to-face interview and an oral health examination. The two interviewers and an examiner who were involved in the data collection were trained and calibrated prior to the survey. The kappa score for intra-examiner reliability was 0.70, while the percentage agreements for the two interviewers ranged between 84% and 92%. The oral examination was carried out with the subject seated on a portable dental chair using a lightweight portable examination light. Teeth were examined using WHO Colour Coded periodontal probe and a disposable mouth mirror.

Subjects were considered to have a NN for prosthodontic treatment when they had missing teeth or if their existing dental prosthesis was ill-fitting or not aesthetically acceptable. As the WHO [16] criteria for assessing prosthodontic need are purposefully not specific about prescribing different types of prosthodontic care [17,18], we followed the ‘*treatment simplification*’ principle (only one type of prosthesis recommended for multiple edentulous spaces in the same person) [19] and based on common practice of prosthodontic care in Malaysia, we considered only removable denture or fixed bridges for replacing missing teeth. A denture was recommended if the anterior or posterior edentulous space was greater than 4 or 2 tooth spaces respectively or the edentulous spaces included a canine and 2 other contiguous teeth or multiple edentulous spaces were involved or there were bilateral edentulous spaces with more than 2 teeth missing and when there was no distal abutment [19]. A bridge was prescribed when there was good periodontal condition with an anterior or posterior space of 4 or 2 teeth (or fewer) respectively and presence of distal abutment.

The SDA model has three components; i) normative need (NN), where treatment needs are assessed by dentists, ii) Impact-Related Need (IRN), where normative needs are combined with OHRQoL and, iii) Propensity-Related Need (PRN), where IRN is combined with the behavioural assessment. In this study, the NN model was compared with the full SDA model [5,6]. The Impact-Related Need (IRN) amongst those who had NN was assessed using the Oral Impacts on Daily Performances index (OIDP) [20]. The OIDP index assessed the impacts of oral conditions on the subjects’ abilities to perform the following daily life activities in the past 6 months: eating, speaking, cleaning teeth or dentures, going out, performing light activities, performing main role, sleeping, smiling, emotional stability and enjoying contact. For each reported oral impact, frequency and severity was assessed using a 5 point Likert scale. The total OIDP scores were calculated by multiplying frequency and severity scores of each performance and then divided with the maximum possible score. As the OIDP index only assesses the ‘ultimate’ oral impacts [19], the cut-off point was set at score 1 to distinguish between people that had an oral impact on their daily life from those that did not. One unique characteristic of the OIDP index is that it allows the reported oral impact to be linked to a specific type of dental treatment required. This feature is called the Condition-Specific OIDP [5]. Subjects who attributed their oral impacts to tooth loss or loose ill-fitting denture/s, were considered as having an Impact-Related Need (IRN) for prosthodontic treatment.

Assessment of Propensity-Related Need (PRN) was done separately for those with a need for dentures or for those needing a bridge because different types of oral health behaviours were taken into account to assess propensity for the different types of prosthesis. If subjects brushed their teeth at least twice a day and had visited a dentist less than two years ago, they were considered as having a high propensity as these two oral health related behaviours were considered important for the success of prosthetic treatment. If they scored poor in any or both of these, for example, if they brushed teeth less than once a day or had an irregular dental visit habit, they were considered as having a low propensity for prosthetic treatment. For bridges, in addition to brushing teeth twice a day and good dental attendance pattern, using fluoride toothpaste and having low sugar intake were measures of high propensity for treatment. If subjects did not conform to the standards set in any of these four behaviours, for example not using a fluoride toothpaste or consuming free sugars more than 4 times a day, they were considered as having a low propensity for bridges.

The proportion of subjects needing prosthodontic care and number of dentures and bridges needed using the NN or SDA models were compared using McNemar test. Then, NN and SDA were compared for the time and numbers of dentists required to provide prosthodontic care for 100,000 Malaysian adults through the Wilcoxon signed rank test. Treatment time estimates were based on those provided by six expert dentists from the Faculty of Dentistry, University of Malaya and the estimation of workforce requirements were made based on the assumption that Malaysian dentists work 1760 hours annually [21].

Finally, three different skill mix models were used to assess differences in workforce requirements when some prosthodontic procedures were delegated. Scenario I (Baseline) represents the current situation in Malaysia where only dentists carry out prosthodontic treatment. In Scenario 2 (Minimum skill mix), prosthodontic PCDs provide only complete dentures, while dentists provide partial dentures and bridges. In Scenario 3 (Maximum skill mix), all denture procedures are delegated to prosthodontic PCDs while dentists only construct bridges. In Malaysia, part time work is not permitted, hence, the annual working hours for both dentists and prosthodontic PCDs are assumed to be similar.

This study was approved by the Ethics Committee of University of Malaya and the University College London Research Ethics Committee.

## Results

Of the 919 eligible employees, 732 agreed to participate (response rate 79.6%). The majority were females (66%) and Malay (83.2%). Their mean age was 41.2 (SD ±7.9) years. About 40% experienced at least one oral impact in the past six months. The mean OIDP score was 2.67 (±6.25). Slightly more than half (55.8%) had a full dentition (having between 28 to 32 teeth) while only 3 (0.4%) were edentulous (Table 1). About 11% had an upper partial denture, less than 2% had either an upper or lower full denture and less than 4% had bridges (Table 2).

**Table 1:** **Sociodemographic characteristics and the prevalence of oral impacts and edentulousness in the sample (n = 732)**

**Variables**		**N**	**%**
Age	30-34 years	214	29.2
	35-44 years	217	29.6
	45-54 years	301	41.2
Gender	Male	249	34.0
	Female	483	66.0
Educational level	Low (primary or secondary school)	433	59.1
	High (Degree/Masters)	299	40.9
Income	≤1500	170	23.2
(Ringgit Malaysia)	1501-3000	371	50.7
	3001-5000	118	16.1
	≥5001	73	10.0
Prevalence of oral impacts		299	40.8
Mean score (±SD) = 2.67(±6.25)			
Range number of teeth present:			
0 (fully edentulous)		3	0.4
1-10 teeth		14	1.9
11-20 teeth		60	8.2
21-27 teeth		247	33.7
28-32 teeth		408	55.8
Mean number of teeth present (±SD) = 26.52(5.28)

**Table 2:** **The percentage of subjects with a dental prosthesis (n = 732)**

**Type of prosthesis**	**N**	**%**
No prosthesis	626	85.5
Has and wore:		
Upper partial denture	84	11.5
Lower partial denture	21	2.9
Upper full denture	12	1.6
Lower full denture	4	0.5
Has but was not wearing:		
Upper partial denture	3	0.4
Lower partial denture	6	0.8
Has at least one bridge on upper jaw	19	2.6
Has at least one bridge on lower jaw	9	1.2

### Prosthodontic treatment need

**Figure 1:**
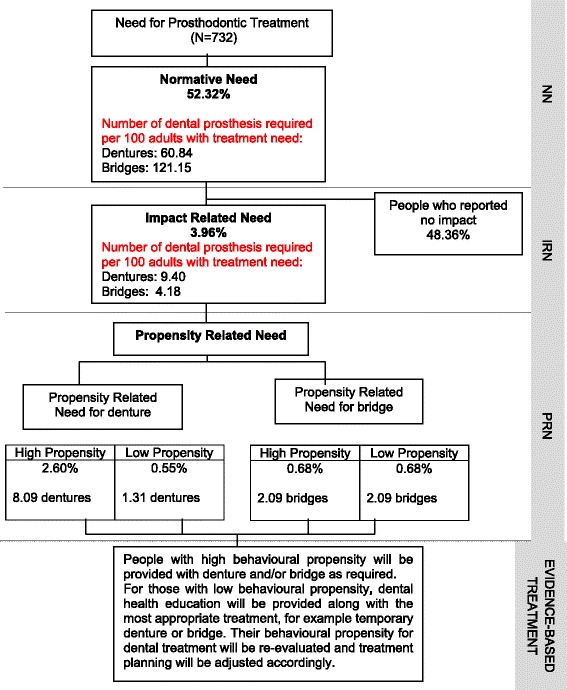
**Comparison of the proportion of sample and the number of denture or bridges required per 100 people with prosthodontics treatment needs using normative and sociodental approaches (N = 732).**

About 52% of subjects had NN for prosthodontic treatment. Of those, less than 4% reported an oral impact attributed to tooth loss or a loose ill-fitting denture. When Propensity-Related Need (PRN) was assessed for those with a NN, only 2.6% and 0.68% had a high behavioural propensity for dentures and bridges respectively. In terms of the number of dentures and bridges needed per 100 people with prosthodontic treatment need, 60.8 dentures and 121.2 bridges were required using NN. This decreased to 8.09 dentures and 2.09 bridges using the SDA approach (Figure 1). Overall, the proportion of subjects needing dentures and bridges was 95.8% and 98.7% lower respectively, when using SDA compared to NN.

### Workforce requirements

Overall, the time needed to provide prosthodontic care based on NN was 173,895 hours per 100,000 Malaysian adults compared to only 4,382 hours using the SDA. The total number of dentists needed for prosthodontic treatment was 98.8 per 100,000 Malaysian adults based on NN compared to 2.49 using SDA (Table 3).

**Table 3:** **Comparison of treatment time (in hours) and the number of dentists required for prosthodontic care per 100,000 adults for normative needs and sociodental needs assessments**

		**Normative need**	**Impact-related need**	**Propensity-related need**	% differences NN-SDA*
Treatment time needed for prosthodontic treatment	Need for dentures	15,425.77	1969.49	1650.73	89.3% (p<0.0001)
	Need for bridges	158,469.94	5464.48	2732.24	98.3% (p<0.0001)
	Total need for dentures and bridges	173,895.71	7433.97	4382.97	97.5% (p<0.0001)
The number of dentists needed for prosthodontic treatment		98.80	4.22	2.49	97.5% (p<0.0001)

*NN = Normative needs, SDA = Sociodental Needs.

The number of dentists needed per 100,000 people for prosthodontic treatment decreased slightly for each skill mix scenario when some prosthodontic procedures were delegated to prosthodontic PCDs using either the NN or SDA model (Table 4). When complete dentures procedures were delegated (Scenario II), the number of prosthodontic PCDs needed was 0.08 as there were very few subjects needing full dentures. If prosthodontic PCDs were allowed to make both complete and partial dentures (Scenario III), 8.76 of them would be needed and the need for dentists decreased from 98.80 to 90.04. Numbers of dentists and PCDs required for prosthodontic care decreased markedly for each skill mix scenario when using the SDA instead of NN. For example, in Scenario III, whereas 90.04 dentists and 8.76 prosthodontic PCDs were needed per 100,000 people using NN, only 1.55 dentists and 0.94 prosthodontic PCDs were needed using SDA (Table 4).

**Table 4:** **The numbers of dentists and denturists needed for prosthodontic care per 100,000 people, assessed using normative and sociodental needs models**

	**The number of dentists or denturists needed for prosthodontic care per 100,000 people**					
	**Scenario 1 (baseline scenario)**		**Scenario II (minimum skill mix)**		**Scenario III (maximum skill mix)**	
Type of model	Dentists	Denturists	Dentists	Denturists	Dentists	Denturists
Normative Need	98.80	0	98.72	0.08	90.04	8.76
Sociodental Need	2.49	0	2.41	0.08	1.55	0.94

## Discussion

This is the first study to compare dental treatment needs and skill mix workforce requirements for prosthodontic care between the Normative Need and the Sociodental Need approaches. The need for prosthodontic treatment was more than 90% lower when SDA was used instead of NN. Although criteria used to assess prosthodontic treatment need were different from other studies on adult or elderly populations [4,6], differences between NN and SDA were similarly large. This may be because the normative need criteria generally recommend replacing all tooth spaces due to missing teeth [22]. That leads to a high prevalence of prosthodontic treatment need. However, only a small proportion of people with oral impacts had impacts related to prosthodontic needs [19]. In the present study, 54.2% had NN but only 4% of them had oral impacts related to missing teeth or ill-fitting denture. The reason for the large differences between normative and sociodental approach may partly be due to the fact that normative assessments do not consider subjective measures of function and oral impacts [1]. NN assessment is based on clinical signs which could appear before any symptoms are experienced, while people are more concerned about the functional and social aspects arising from oral diseases that might affect their daily performances [23]. In prosthodontics care, loss of teeth may not lead people to seek for treatment if they are free of pain and are satisfied with their function and aesthetics.

When prosthodontic PCDs were considered in estimating dental workforce requirements, the number of dentists required decreased slightly for all the skill mix scenarios. The reason for this is because our samples were adults aged between 30–54 years old. Most of them had oral conditions that would benefit more from dental bridges that are only done by dentists. The skill mix scenario used in this study is modified from Gallagher and colleagues [12]. Their study also showed that the number of dentists needed to meet the future needs of the elderly in England decreased when skill mix approach is used. The number of PCDs required depends on how much care dentists are willing to delegate. However, despite evidence showing the benefit of using skill mix, the acceptance of working as a team in dentistry has not been overwhelming [8]. In Malaysia, skill mix has been introduced in periodontology, orthodontics, oral surgery and paedodontics. Expanded-duty PCDs who received further training in their specialized area are allowed to do simple tasks such as scaling, root planning and issuing of removable orthodontic appliance to both children and adults patients. In line with this, prosthodontic PCDs should be introduced into the skill mix. Dentists should focus on general diagnosis and perform complex prosthodontic treatment such as bridges and complex denture cases, supported by PCDs who provide basic care, uncomplicated denture cases and prevention of oral diseases. The incorporation of skill mix approach in prosthodontics care should improve access of care especially for low income populations and in rural settings

The SDA reflects the populations’ actual dental needs and their ability to achieve maximum health gain. The inclusion of subjective measures of need should complement the normative approach and allow for the consideration of biological, psychological and socio-environmental factors [24]. In addition, subjective measures also have the potential to better predict use of health services and provide a more accurate projection of workforce needs [25]. Estimates of need should be based only on interventions that lead to oral health gains. People who have good oral health behaviour should be given priority in receiving treatment as their treatment outcome will be better. People having low propensity relating to dental interventions should be given health education to change their behaviours to a level appropriate to their treatment need. Despite the significant implications of the SDA and skill mix approaches for workforce estimation and planning, it may not be achievable in practice because of some constraints. For example, retraining of dentists will be needed to implement the SDA. In addition, it may not always be practical to use skill mix in small and dispersed clinics compared to centralized settings. The funding mechanism, the public or private mix provision and the country’s labour market factor could also affect the practicality of the approach.

This study has some limitations. This study was conducted on an adult population that is not fully representative of the population of Malaysia. Different findings could be obtained if non-working adults or adults living in rural areas were included. The samples were adults aged between 30–54 years, so there could be an underestimation of the prevalence of tooth loss in the adult population as only younger working adults were included. In addition, time estimates for prosthodontic work were based on experts’ opinions and therefore they may not be precise. However, the experts made the estimates based on timings data obtained from an observation study conducted in various private and public dental clinics. This should provide a reasonable estimate of time needed for prosthodontic work under local circumstances. Because of the lack of representativeness of the samples, the generalization of the findings to the whole population is limited. However, the large size of the differences between NN and SDA found in this study illustrate the gap between NN and SDA and apply to assessments of dental needs in all populations.

## Conclusions

Using the sociodental approach resulted in much lower estimates of dental need and workforce requirements for prosthodontic treatment than using the normative method. When skill mix approach is used, the need for dentists decreased further. The estimation of dental workforce requirements using the sociodental approach provide a more realistic estimate as it is based on normative and impact related needs of a population. The use of skill mix in dentistry would enhance delivery of dental care. Future studies using the sociodental and skill mix approaches on nationally representative samples should provide relevant information for policymakers and planners at a national level.

## Abbreviations

NN: Normative need
SDA: Sociodental approach
OIDP: Oral impacts on daily performances
OHRQoL: Oral health related quality of life
PCD: Professionals complementary to dentistry
IRN: Impact-related need
PRN: Propensity-related need
CS: Condition-specific
